# A case of synchronous double cancers consisting of maxillary gingival carcinoma and intraductal papillary mucinous carcinoma, invasive: case report

**DOI:** 10.1186/s12903-023-03253-y

**Published:** 2023-08-26

**Authors:** Ryosuke Iwama, Hitoshi Miyashita, Atsumu Koketsu, Kiyoshi Kume, Fumiyoshi Fujishima, Atsushi Masamune, Tetsu Takahashi

**Affiliations:** 1 Division of Oral and Maxillofacial Surgery, Tohoku University Graduate School of Dentistry, Sendai, Japan; 2 Division of Gastroenterology, Tohoku University Graduate School of Medicine, Sendai, Japan; 3 Department of Pathology, Tohoku University Hospital, Sendai, Japan

**Keywords:** Synchronous double cancers, Maxillary gingival carcinoma, Intraductal papillary mucinous neoplasm

## Abstract

**Background:**

The development of synchronous multiple primary cancers is one of the major causes of death in patients with head and neck cancer. Herein, we report a case of synchronous intraductal papillary mucinous carcinoma (IPMC), invasive in a patient with maxillary gingival carcinoma.

**Case presentation:**

A 73-year-old female visited our hospital complaining of a mass on the left side of the maxillary gingiva. Intraorally, an exophytic tumor, 50 × 25 mm in size, was found on the gingiva of the left maxillary posterior, and a diagnosis of squamous cell carcinoma was revealed by cytology. Emission tomography/ computed tomography with 18 Fluorodeoxyglucose-Positron (18FDG- PET/ CT) showed increased accumulation in the left maxillary gingiva, the left side of cervical lymph nodes, and the main pancreatic duct. The pancreatic ductal tumor was performed the biopsy at esophagogastroduodenoscopy (EGD) and resulted in a pathological diagnosis of IPMC, invasive. The patient was diagnosed as synchronous double primary cancers consisting of maxillary gingival carcinoma cT4aN2bM0 and IPMC, invasive cT3N0M0. She refused radical treatment, and died 11 months later.

**Conclusion:**

18FDG- PET/ CT, EGD and multidisciplinary approach is required for the detection and determining the treatment strategy of synchronous double primary cancers.

## Background

Head and neck squamous cell carcinoma (HNSCC) is the sixth most prevalent cancer in the world [1], with a high rate of development of multiple primary carcinomas [2]. Multiple primary cancers were defined by Warren et al. as the following: 1) each tumor shows a certain malignant image, 2) each tumor is located in mutually distant sites, and 3) one is not a metastasis of the other [3]. Moertel et al. defined multiple primary cancers as being synchronous if they are detected within 6 months of the primary cancer [4]. The presence of synchronous multiple primary cancers is a major clinical problem and a leading cause of death in patients with head and neck cancer [5].

It has been reported that synchronous multiple primary cancers are highly prevalent in the head and neck region, upper gastrointestinal tract, and lungs but rare in the pancreas [6, 7]. Pancreatic carcinoma includes the following histological subtypes: pancreatic ductal adenocarcinoma, intraductal papillary mucinous carcinoma (IPMC), invasive, acinar cell carcinoma, neuroendocrine carcinoma. Among these subtypes, adenocarcinoma contributes to about 85% of primary pancreatic malignancies. IPMNs are characterized by papillary growths in the pancreatic ducts that produce mucus. They can be benign or progress to become malignant during the course of the disease, leading to high-grade dysplasia (HGD) or IPMC, invasive. Depending on the location of origin, IPMNs can be classified as main pancreatic ductal (MD-IPMN), branched ductal (BD-IPMN), or mixed types. The Fukuoka consensus criteria provide different parameters for surgical resection according to the classification of the IPMNs [8, 9].

Here we report a case of synchronous multiple primary cancers with maxillary gingival cancer and MD-IPMC.

## Case presentation

**Fig. 1:**
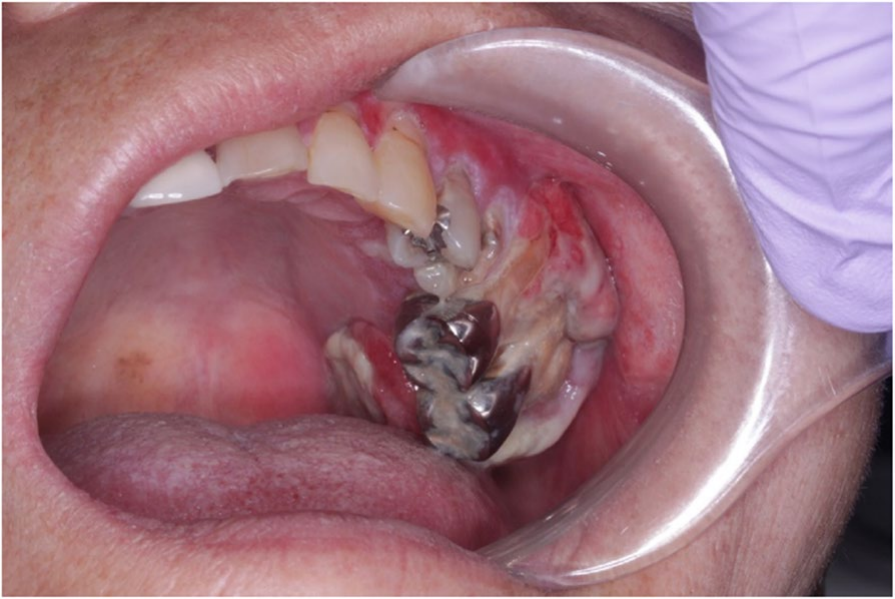
Intraoral photograph showing an exophytic tumor with a rough surface on the left maxillary gingiva

**Fig. 2:**
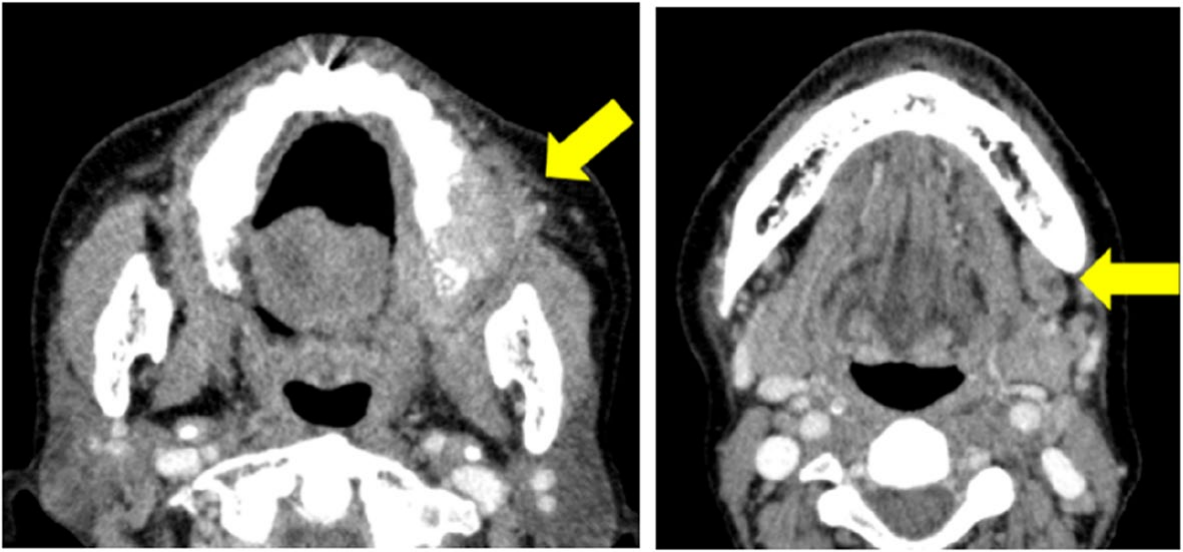
CT image showing bone destruction and enlarged Level 1B lymph node due to left lateral maxillary gingival tumor

**Fig. 3:**
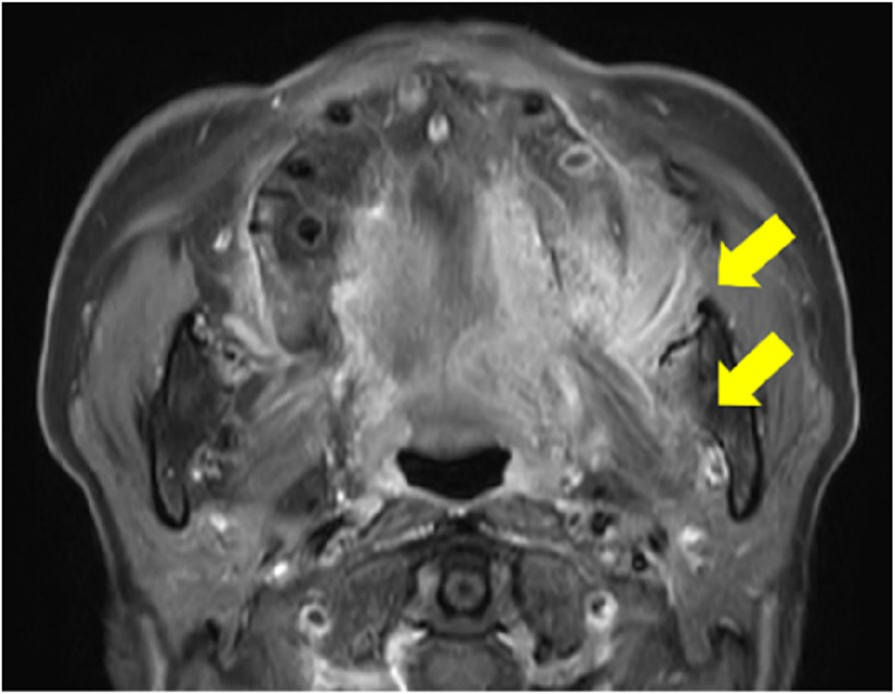
MR image showing a mass in the left maxillary gingiva extending to the base of the pterygoid process and the masticatory muscle space

**Fig. 4:**
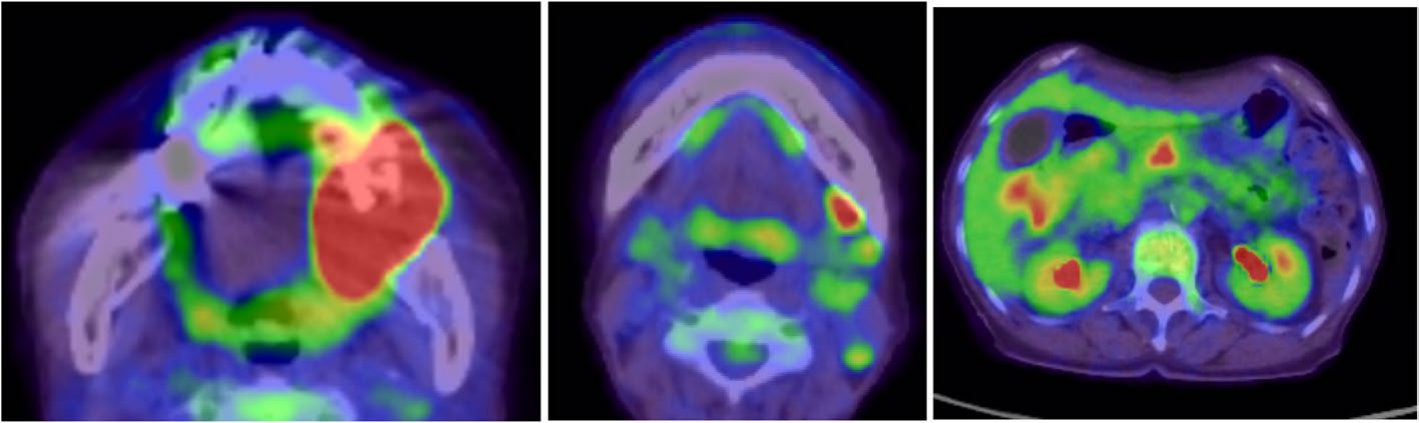
18FDG-PET/CT image showing hyper-accumulation in the left lateral maxilla (SUVmax 32.5), left lateral cervical lymph node (SUVmax 4.5–18.5), pancreatic head (SUVmax 6.2), and pancreatic body (SUVmax 7.4)

**Fig. 5:**
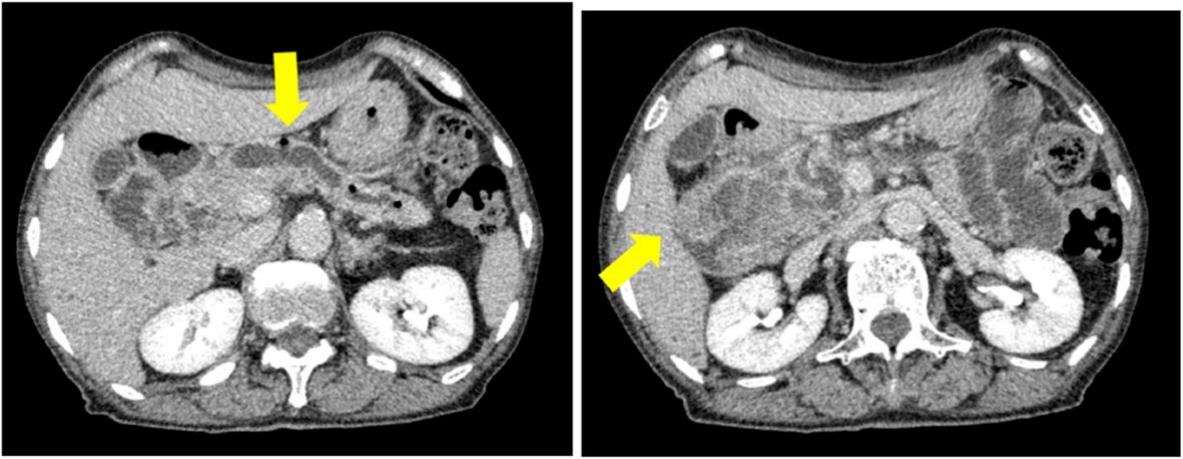
CT image showing the main pancreatic duct in the pancreatic body dilated to 15 mm in diameter and a tumor in the pancreatic head in contact with the duodenum

**Fig. 6:**
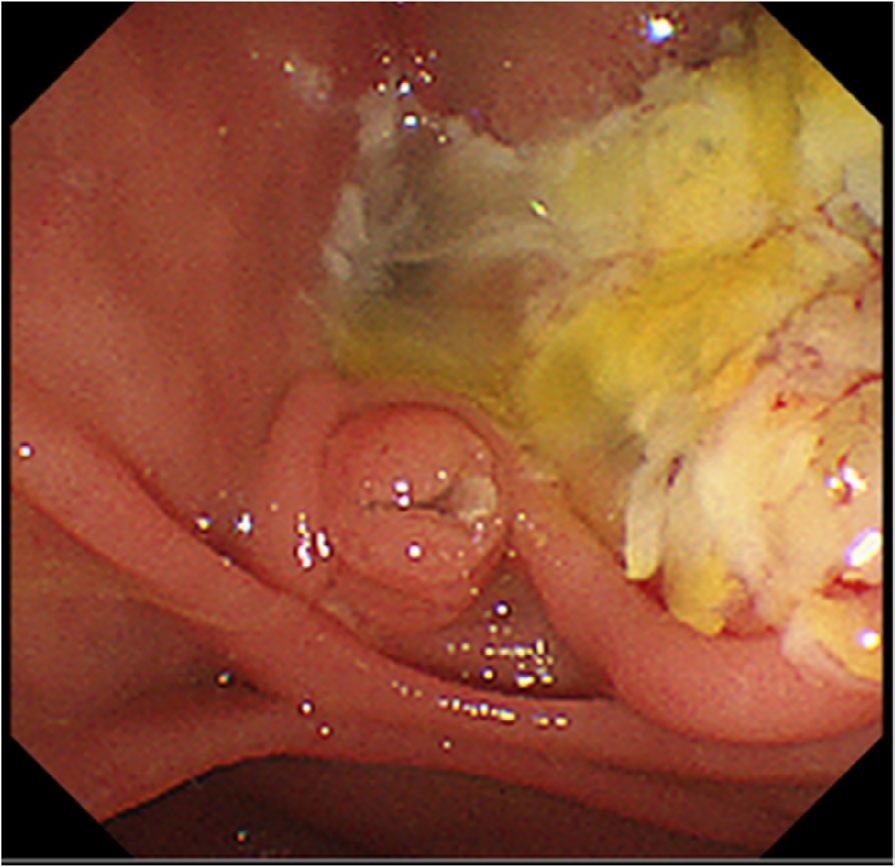
Endoscopic image showing a tumor perforating the intestinal wall at a site near the Vater papilla

**Fig. 7:**
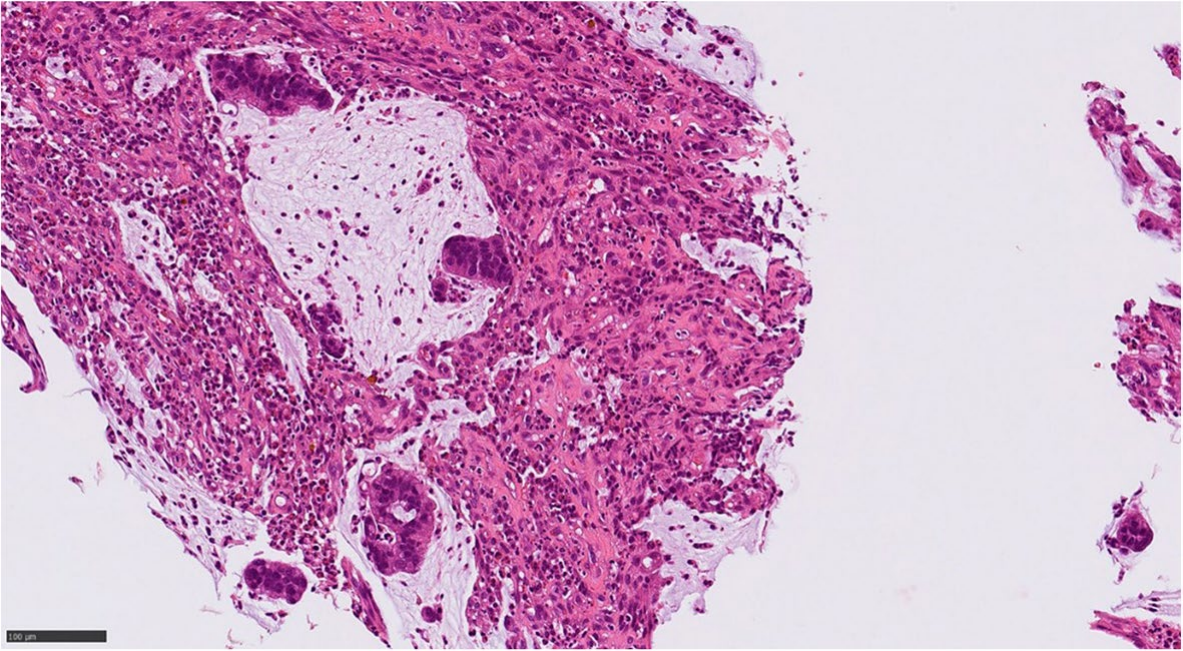
Clusters of tumor cells were floating in the mucus lake, sometimes accompanied by glandular formation

**Fig. 8:**
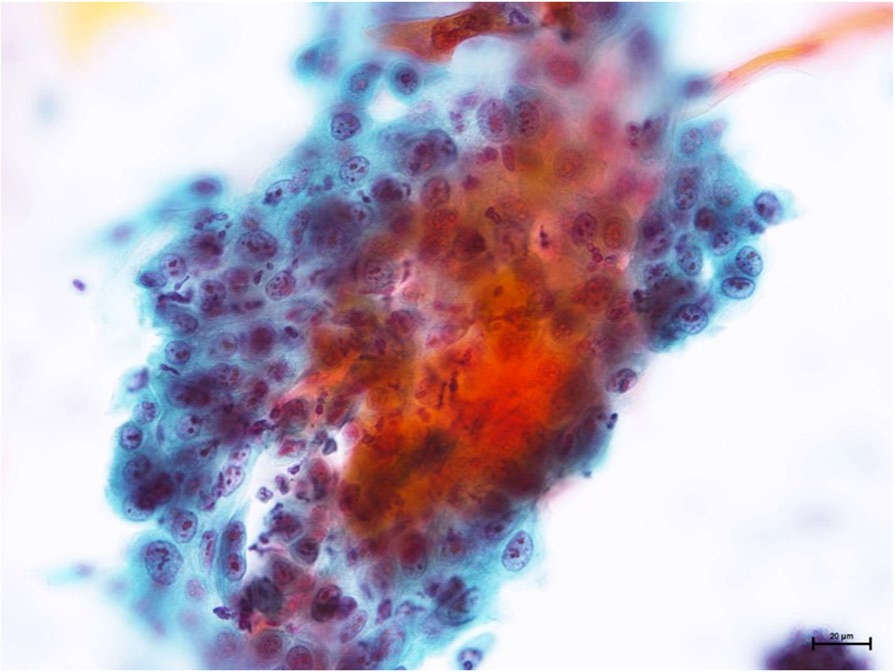
The tumor cells demonstrated irregularly shaped nuclei and a high nucleus/cytoplasm ratio (Papanicolaou stain)

A 73-year-old female visited a local dental clinic complaining of a mass on the left side of the maxillary gingiva and was referred to our hospital in August 2020. Although the patient reported an unremarkable medical history, a blood test revealed an HbA1c of 6.9%, suggesting a diagnosis of diabetes mellitus. Extraorally, slightly enlarged lymph nodes were palpable bilaterally in the neck. Intraorally, an exophytic tumor, 50 × 25 mm in size, was found on the gingiva of the left maxillary posterior (Fig. 1). Contrast-enhanced computed tomography (CT) showed a mass with irregular bony destruction and contrast effects in the left side of the maxilla, extending to the base of the pterygoid muscle and the pterygoid process. Lymph nodes with internal defects were found in the left level IB and IIA area, suggesting multiple lymph node metastasis. However, there were no evidence of extranodal extension due to the defined marginal morphology. (Fig. 2). Contrast-enhanced magnetic resonance imaging (MRI) showed a mass in the left maxillary gingiva extending beyond the maxillary nodule to the base of the pterygoid process and the masticatory muscle space (Fig. 3). Emission tomography/ computed tomography with 18 Fluorodeoxyglucose-Positron (18 FDG-PET/CT) showed increased accumulation in the left maxilla (SUVmax 32.5) and the level IB (SUVmax 18.5) and level IIA (SUVmax 4.2) left cervical lymph nodes. In addition, there was an increased accumulation in the head of the pancreas (SUVmax 6.2) and the body of the pancreas (SUVmax 7.4), accompanied by a dilatation of the main pancreatic duct (MPD) (Fig. 4). No metastasis to the paratracheal lymph nodes or other organs was detected. The patient was referred to the Department of Gastroenterology for a detailed examination of the pancreatic tumor, and contrast-enhanced abdominal CT and MRI examinations were performed. A neoplastic lesion with a maximum diameter of 82 mm was observed in the pancreatic head. The tumor was in contact with the Vater papilla and the descending leg of the duodenum; it was suspected that the tumor had invaded the duodenum (Fig. 5). Esophagogastroduodenoscopy (EGD) showed that the tumor had indeed invaded the descending duodenum and perforated the intestinal wall (Fig. 6); a specimen was collected from the same area. Histopathologically, the diagnosis was mucinous carcinoma (Fig. 7). In combination with the imaging findings, the final diagnosis was cT3N0M0, StageIII MD-IPMC. On the other hand, cytodiagnosis of the tumor in the maxillary gingiva revealed SCC (Fig. 8), with a final diagnosis of cT4bN2bM0, Stage IVB SCC, based on the staging described by the 8th edition of Unio Internationalis Contra Cancrum.

We explained the necessity of histopathological examination for treatment planning and proposed resection of the left maxilla and radical neck dissection followed by resection of the pancreatic tumor, but the patient refused this treatment plan; thus, we pursued a supportive care strategy. The patient underwent palliative irradiation of 30 Gy/10Fr in the oral cavity and was subsequently followed up at another hospital. She died 11 months after her initial diagnosis due to respiratory failure caused by multiple lung metastases.

## Discussion and conclusions

HNSCC patients present with a 5.3% occurrence of synchronous multiple cancers [10]. Based on the concept of “field cancerization”, HNSCC is considered a risk factor for multiple primary cancers in the head and neck, lung, and esophagus [11]. A previous review of HNSCC reported that the cumulative incidence of heterogeneous second primary cancers was 7.2% at six months, 17.9% at five years, and 23.1% at 10 years [7]. In this case, IPMC was found as a synchronous second primary cancer. The occurrence of synchronous cancer of the pancreas in patients with head and neck carcinoma is exceedingly rare, and to our knowledge, cases of multiple primary cancers with maxillary gingival carcinoma and MD-IPMC have not been reported. 18FDG-PET/CT and EGD are useful tools in the detection of multiple primary cancers in HNSCC patients. The sensitivity and specificity of 18FDG-PET/CT in the search for distant metastases and multiple cancers in HCSCC patients are very high, at 0.888 and 0.971, respectively [12]. The sensitivity of 18FDG-PET/CT in the detection of pancreatic cancer is also high, at 89.1% [13]. Even when limited to IPMNs, 18FDG-PET/CT has a sensitivity and specificity of 96.8 and 91.1%, respectively, demonstrating significant utility as a diagnostic tool [14]. Kamegaka et al. reported a triple cancer case; cancers in the middle part of the extrahepatic bile duct, the pancreas head, and the supraglottis [15]. The case was diagnosed with supraglottic carcinoma with hoarseness as the main complaint, and subsequently 18FDG-PET/CT examination was performed, which revealed the extrahepatic bile duct cancer and the pancreas head cancer. In a study comparing the frequency of detection of multiple cancers by 18FDG-PET/CT and EGD, 18FDG-PET/CT showed superior results, but it is considered that both are necessary because early esophageal and gastric cancers are difficult to be detected by 18FDG-PET/CT due to low FDG uptake [16, 17].

The frequency of invasive carcinoma or HGD in MD-IPMN is 61.6% (range, 36–100%) and the frequency of invasive carcinoma is 43.1%, while the mean frequency of invasive carcinoma or HGD in BD-IPMN is 25.5% (range, 6.3–46.5%) and the mean frequency of invasive carcinoma is 17.7% (range 1.4–36.7%) [8, 9]. In the case of MD-IPMN, surgical resection is highly recommended in the presence of high-risk stigmata such as MPD dilation over 10 mm, cystic lesions of the pancreatic head with obstructive jaundice, and the presence of a solid component with the contrast-enhancement [8, 9]. MPD dilation of 5–9 mm is considered to be a "worrisome feature" warranting close examination and follow-up rather than immediate resection [8, 9]. Although the treatment strategy differs depending on the site of origin, tumor diameter, invasion of surrounding tissues, and presence or absence of lymph node metastasis in the pancreas, patients may undergo pancreaticoduodenectomy, caudal pancreatectomy, or total pancreatectomy. For IPNC that are surgically resected at an early stage (T1N0), the 5-year survival rate is about 40%, and for IPMC that are more advanced or have lymph node metastases, the survival rate is about 20% [18]. This is comparable to the 5-year survival rate of about 27% in pancreatic adenocarcinoma patients who underwent surgical resection and postoperative adjuvant chemotherapy [19]. Although the mechanism is unknown, it has been suggested that the occurrence and malignancy risk of IPMN are related to diabetes mellitus, and this case also showed diabetes mellitus [20, 21]. In addition to diabetes mellitus, family history in the first degree, chronic pancreatitis, and history of insulin use have also been associated as risk factors, and these cases require careful follow-up.

In this case, the patient had a maxillary gingival carcinoma corresponding to T4bN2b in the head and neck region. Previous studies have reported 5-year survival rates of 25.4–41.0% for T4 patients who underwent surgery and 20.5–36.9% for those with positive cervical lymph nodes [22–25]. Although a multidisciplinary approach is required in determining the treatment strategy for patients with synchronous multiple cancers, treatment decisions should be guided on the following three principles [26]:

1. Give priority to high-grade cancers.
2. Treat more advanced stages of cancer first. If it is difficult to cure the more advanced cancer, the secondary cancer may not warrant treatment.
3. Simultaneous treatment should be completed when possible.

Treatment outcomes have been reported in cases of head and neck cancer with synchronous esophageal carcinoma [27, 28]. Panosetti et al. have shown that synchronous carcinoma has a lower survival rate than metachronous carcinoma (18% vs. 55%), and radical or curative treatment of head and neck cancer cases complicated by IPMC, invasive is extremely difficult [29]. In this case, the maxillary gingival carcinoma was more advanced than the IPMC; hence, resection of the left maxilla and radical neck dissection followed by resection of the pancreatic carcinoma was treatment planned, but the patient declined and requested palliative care only. Although the prognosis of IPMC, invasive is better than that of pancreatic adenocarcinoma, the prognosis of stage IVB maxillary gingival cancer is poor.

In conclusion, it is important to identify synchronous multiple primary carcinomas in the treatment of HNSCC, and 18FDG-PET/CT is quite useful. Furthermore, it follows that a multidisciplinary approach is needed for these cancers.

## Data Availability

We include all data supporting the findings in the manuscript.

## Abbreviations

HNSCC: Head and neck squamous cell carcinoma
IPMNs: Intraductal papillary mucinous neoplasms
IPMC: Intraductal papillary mucinous carcinoma
HGD: High-grade dysplasia
CT: Computed tomography
MRI: Magnetic resonance imaging
18FDG-PET/CT: Emission tomography/ computed tomography with 18 Fluorodeoxyglucose-Positron
MPD: Main pancreatic duct
EGD: Esophagogastroduodenoscopy
